# Review of Poliovirus Transmission and Economic Modeling to Support Global Polio Eradication: 2020–2024

**DOI:** 10.3390/pathogens13060435

**Published:** 2024-05-22

**Authors:** Kimberly M. Thompson, Kamran Badizadegan

**Affiliations:** Kid Risk, Inc., Orlando, FL 32819, USA; kb@kidrisk.org

**Keywords:** polio, eradication, modeling, policy, decision

## Abstract

Continued investment in the development and application of mathematical models of poliovirus transmission, economics, and risks leads to their use in support of polio endgame strategy development and risk management policies. This study complements an earlier review covering the period 2000–2019 and discusses the evolution of studies published since 2020 by modeling groups supported by the Global Polio Eradication Initiative (GPEI) partners and others. We systematically review modeling papers published in English in peer-reviewed journals from 2020–2024.25 that focus on poliovirus transmission and health economic analyses. In spite of the long-anticipated end of poliovirus transmission and the GPEI sunset, which would lead to the end of its support for modeling, we find that the number of modeling groups supported by GPEI partners doubled and the rate of their publications increased. Modeling continued to play a role in supporting GPEI and national/regional policies, but changes in polio eradication governance, decentralized management and decision-making, and increased heterogeneity in modeling approaches and findings decreased the overall impact of modeling results. Meanwhile, the failure of the 2016 globally coordinated cessation of type 2 oral poliovirus vaccine use for preventive immunization and the introduction of new poliovirus vaccines and formulation, increased the complexity and uncertainty of poliovirus transmission and economic models and policy recommendations during this time.

## 1. Introduction

The over 100 publications related to polio eradication modeling since 2020 [1,2,3,4,5,6,7,8,9,10,11,12,13,14,15,16,17,18,19,20,21,22,23,24,25,26,27,28,29,30,31,32,33,34,35,36,37,38,39,40,41,42,43,44,45,46,47,48,49,50,51,52,53,54,55,56,57,58,59,60,61,62,63,64,65,66,67,68,69,70,71,72,73,74,75,76,77,78,79,80,81,82,83,84,85,86,87,88,89,90,91,92,93,94,95,96,97,98,99,100,101,102] provide an indication of ongoing challenges in the quest to achieve the 1988 World Health Assembly (WHA) resolution to eradicate paralytic poliomyelitis (“polio”) [103]. The WHA target of achieving polio eradication by the year 2000 led a small group of core partners to establish the Global Polio Eradication Initiative (GPEI) to provide, mobilize, and coordinate the necessary planning, financial, and technical resources [104]. Although early action plans expected the achievement of polio eradication as a result of national efforts to strengthen their routine polio immunization programs and global improvements in surveillance, the strategies for meeting the year 2000 target also included immunization campaigns [105]. Over time, operational challenges and the complexity of polio eradication became increasingly apparent, with successive target deadlines for various objectives repeatedly missed [104]. Shortly before 2000, policymakers began to recognize the potential role of using insights from dynamic poliovirus transmission (e.g., [106,107,108,109]) and static health economics (e.g., [110]) modeling to support decision-making for the polio eradication endgame. These early studies helped to motivate the GPEI core partners to formally engage independent modeling groups to provide policy and decision insights, as discussed in a prior systematic review of poliovirus transmission and health economic modeling studies published in English from 2000 to 2019 [1]. 

Recognizing the importance of facilitating access for modelers to national, regional, and global data, the GPEI core partners developed a data sharing agreement to support polio modeling groups [1]. The prior review of 2000–2019 included documentation of all polio-related publications by the three independent modeling groups supported by GPEI core partners under the data sharing agreement as of 2019 [1]. Since 2019, however, the polio modeling landscape has evolved and expanded. While two groups, Kid Risk, Inc. (KRI, Orlando, FL, USA) and Imperial College (IC, London, UK), continued to perform independent modeling with support from GPEI partners, in 2020, the third modeling group included in the prior review [1], the Institute for Disease Modeling (IDM, Seattle, WA, USA), became part of the Bill and Melinda Gates Foundation (BMGF). With this transition, IDM modeling went fully under the operational control of an influential GPEI core partner that presumably could rely on internal modeling results for decision-making without external peer review. In addition, with personnel moves and the expansion of the GPEI data sharing agreement to allow each core partner to support more than one independent modeling group, several other groups also became GPEI-partner-supported polio modeling groups. These include the London School of Hygiene and Tropical Medicine (LSHTM), the South African Centre for Epidemiological Modelling and Analysis (SACEMA), and the Georgia Institute of Technology (GIT). In addition to studies by GPEI-supported polio modeling groups (henceforth “modeling groups”), the literature continues to include some poliovirus transmission and economic modeling studies published by others.

The complexity of modeling poliovirus transmission remains a challenge due to the presence of three distinct poliovirus serotypes (i.e., types 1, 2, and 3) and numerous strains of live polioviruses (LPVs), including wild polioviruses (WPVs), live attenuated oral poliovirus vaccines (OPVs), and OPV-related strains. OPV-related strains include vaccine-derived polioviruses (VDPVs) that can result in outbreaks of circulating VDPVs (cVDPVs, also referred to as “variant” polioviruses), if OPV-related viruses transmit in populations with low immunization coverage. The GPEI reports cases of WPVs and cVDPVs weekly, but not other strains. However, other OPV-related strains, including immunodeficiency-associated VDPVs (iVDPVs), which occur rarely in individuals with primary immunodeficiencies, represent an important concern for eradication because immunodeficient patients can become prolonged or chronic poliovirus excreters and potentially act as a local source for re-introduction in an otherwise polio-free population. OPV-related risks also include rare cases of vaccine-associated paralytic polio (VAPP) that can occur in OPV recipients or their close contacts. 

Additional challenges arise from the complex historical use of inactivated poliovirus vaccines (IPV) and OPV, including different formulations of OPV. Historically, OPVs used Sabin OPV strains, for example, trivalent OPV (tOPV, containing types 1, 2, and 3), monovalent OPV (mOPV, containing type 1, 2, or 3, and identified as mOPV1, mOPV2, or mOPV3), or bivalent OPV (bOPV, containing types 1 and 3). More recently, however, the development of a novel type 2 OPV (nOPV2) strain led to its use instead of or in addition to mOPV2 in outbreak response immunization campaigns, with novel OPV types 1 and 3 rapidly being developed. In addition, while all IPV formulations remain trivalent, licensed vaccines use either wild seed strains (Salk IPV) or Sabin OPV seed strains (Sabin IPV), and delivery of IPV can occur standalone in full or fractional dosing or in combination with other antigens. All national immunization programs deliver poliovirus vaccines in their routine immunizations (RI) according to age-based national schedules. Some countries also deliver poliovirus vaccines in supplemental immunization activities (SIAs), which target individuals within a specific age range over a short period of time, either as planned preventive SIAs (pSIAs) or reactive outbreak response SIAs (oSIAs). Poliovirus modeling studies must ideally incorporate all relevant biological, epidemiological, risk, and economic data on different poliovirus types and strains to provide value to policymakers. 

Since the body of literature has expanded significantly since the prior review [1], we sought to document the publications of the different modeling groups and to systematically review poliovirus transmission and economic modeling results published in English for 2020–2024.25. The prior review noted the absence of a single repository for global polio policy decisions [1], and this remains an ongoing challenge. For this update, we recognized the opportunity to review the GPEI annual reports, conclusions and recommendations of the World Health Organization Strategic Advisory Group of Experts on Immunization (SAGE) meetings, and reports of technical advisory groups to assess the impact of modeling in supporting polio policies and decisions. This review seeks to provide insights into the role and impact of modeling on national, regional, and global policies with respect to the ongoing polio endgame.

## 2. Materials and Methods

**Figure 1:**
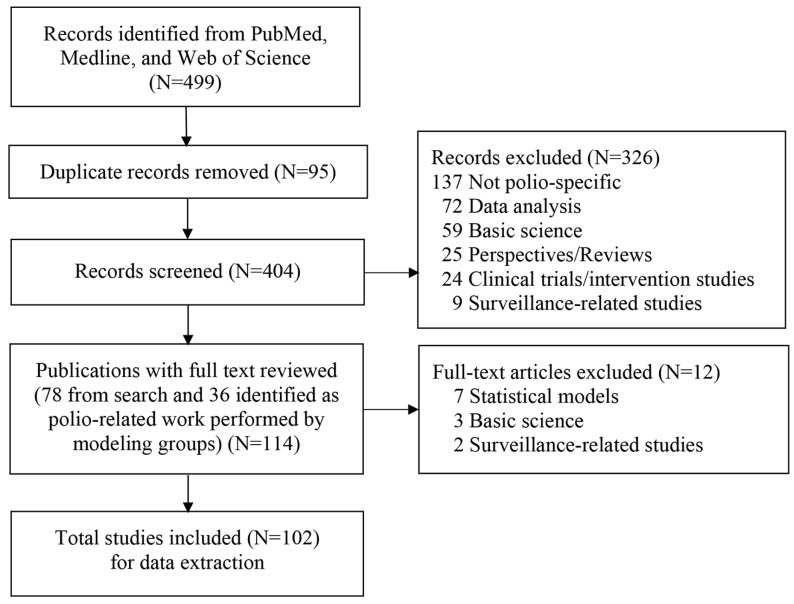
Literature search process.

For each of the 102 studies that met our inclusion criteria [1,2,3,4,5,6,7,8,9,10,11,12,13,14,15,16,17,18,19,20,21,22,23,24,25,26,27,28,29,30,31,32,33,34,35,36,37,38,39,40,41,42,43,44,45,46,47,48,49,50,51,52,53,54,55,56,57,58,59,60,61,62,63,64,65,66,67,68,69,70,71,72,73,74,75,76,77,78,79,80,81,82,83,84,85,86,87,88,89,90,91,92,93,94,95,96,97,98,99,100,101,102] (Figure 1), we used the same categories and hierarchy to classify the type of modeling study as in the prior review [1]: (i) integrated modeling (i.e., including both dynamic transmission and economic modeling), (ii) dynamic transmission models, subcategorized as differential-equation-based (DEB), stochastic compartmental (SC), individual-based (IB), and/or discrete event simulation (DES), (iii) economic analyses, or (iv) other. The “other” category (i.e., polio publications not reporting poliovirus transmission or health economic modeling) applies only to the modeling groups, which we categorized as: statistical analyses (further classified as risk assessment, vaccine effectiveness, or mucosal immunity studies), reviews, discussions of policy options, or perspectives/commentaries as before [1]. 

In this review, we provide a synopsis of all poliovirus-related publications by the six modeling groups as of early 2024 and all poliovirus transmission and economic modeling studies identified in our search and published between 2020–2024.25. We also summarize trends in the numbers and types of polio-related papers published by the modeling groups and other poliovirus transmission and economic modeling papers, for which we update the summary statistics from the prior review [1]. To appropriately discuss trends, we updated the attribution of studies published 2000–2019 [1] for 2 studies led by an LSHTM researcher (Dr. Kathleen O’Reilly) with IC coauthors [111,112] from IC to LSHTM. Assuming that the GPEI data sharing agreement with LSHTM could cover other polio researchers at LSHTM, in our analysis of trends, we changed the attribution of a 2001 transmission modeling study by Dr. Paul Fine [113] from the “other” transmission modeling group in prior review [1] to LSHTM. We also added other LSHTM polio-related publications between 2000 and 2019 (i.e., statistical analyses, vaccine effectiveness and immunology studies, reviews, and perspectives [114,115,116,117,118,119,120,121,122,123,124,125,126,127]), although we note the continued exclusion of poliovirus transmission modeling papers published by these LSHTM researchers prior to 2000 [106,107]. The shift of IDM into BMGF created challenges for characterizing the contributions of IDM and BMGF modelers. We searched for and included polio publications by IDM named- staff identified in the prior review still at IDM/BMGF (i.e., Drs. Guillaume Chabot-Couture, Michael Famulare, Steve Kroiss, Hil Lyons, and Kevin McCarthy) [1]. Similar to the prior review [1], which mentioned but did not include a 2017 BMGF-led statistical analysis that characterized population immunity in the Democratic Republic of the Congo [128], this updated review excludes results from serological surveys [129,130] and a statistical approach for analyzing and presenting polio surveillance data to supplement standard performance indicators [131]. 

In addition to characterizing trends in the numbers and types of publications, we identified papers that covered the same themes identified in the prior review [1]. We also added themes that emerged between 2020 and 2024, including nOPV, COVID-19/pandemic modeling, secondary effects of OPV, GPEI transition and integration, and containment risks. For the subset of studies that included poliovirus transmission modeling, we identified the specific population modeled.

As noted in the prior review [1], no depository of polio policy or GPEI decisions exists, which undermines efforts to systematically review and document the decision-support provided by modeling. However, while national, regional, and global health leaders and the GPEI leaders make policy decisions that SAGE does not review, for this analysis, we reviewed the published conclusions and recommendations from SAGE biannual meetings from inception in 1999 through April 2024 [132]. We found that SAGE discussions occasionally referred to input from technical advisory groups, which led us to review all available reports from the Advisory Committee on Polio Eradication (ACPE), which met nine times during 2004–2009, and the SAGE polio working group (SPWG), which met 27 times since its creation in 2008 through April 2024. From SPWG meeting notes for the record, we extracted the model group affiliations for SPWG meeting attendance. SPWG membership included Dr. Kimberly Thompson from KRI (term, meetings 1–14, 2008–2017), Dr. Nicholas Grassly from IC (meetings 1–19, 2008–2020), Dr. Guillaume Chabot-Couture from IDM/BMGF (meetings 15–27, 2017–present), and Dr. Kathleen O’Reilly from LSHTM (meetings 20–27, 2020–present). We identified participation in SPWG meetings by representatives of the groups noting participation as a SPWG member (M) or guest (G), and we noted whether a representative of the modeling group presented its work during the meeting or if some other speaker presented work from the modeling group (e.g., another modeling group or a GPEI partner). Finally, we also reviewed the 1999–2022 GPEI Annual Reports [133,134,135,136,137,138,139,140,141,142,143,144,145,146,147,148,149,150,151,152,153,154,155,156]. From all of the available reports, we searched the references to the modeling groups, including citations of their studies, and we extracted the context and nature of the modeling mentioned. 

With changes in GPEI leadership (Dr. Bruce Aylward (before 2000–2014) [157], Dr. Hamid Jafari (2015) [158], Mr. Michel Zaffran (2016–2020) [159] and Mr. Adian O’Leary (2021–present) [159]), we observed that GPEI changed its governance structure, and we separately reported how GPEI strategies and timelines evolved over time [104]. These changes included the creation of the GPEI Strategy Committee in December 2014 [157]. From experience, we know of modeling presentations made to the GPEI Strategy Committee (e.g., KRI, IC, and IDM/BMGF all presented modeling related to responding to the global situation with type 2 outbreaks in April and May 2022), but in the absence of published meeting records or minutes, we could not report on the role of modeling input in GPEI Strategy Committee decisions. 

## 3. Results

This section begins with a brief overview of each of the 102 studies identified by the search process summarized in Figure 1 [1,2,3,4,5,6,7,8,9,10,11,12,13,14,15,16,17,18,19,20,21,22,23,24,25,26,27,28,29,30,31,32,33,34,35,36,37,38,39,40,41,42,43,44,45,46,47,48,49,50,51,52,53,54,55,56,57,58,59,60,61,62,63,64,65,66,67,68,69,70,71,72,73,74,75,76,77,78,79,80,81,82,83,84,85,86,87,88,89,90,91,92,93,94,95,96,97,98,99,100,101,102]. As before [1], we organize these by modeling group (i.e., KRI, IC, LSHTM, IDM/BMGF, SACEMA, GIT), and others. We then provide summary statistics of the included publications and characterize overall trends and themes, considering these in the context of the prior review [1]. Finally, we report the apparent impact of modeling in GPEI Annual Reports and SAGE conclusions and recommendations.

### 3.1. Studies That Met Inclusion Criteria Published 2020–2024.25

#### 3.1.1. KRI

In 2020, KRI published reviews on several topics, including the prior review of modeling studies [1], potential use of vaccine patches for immunization, including IPV [2], and the performance of the United States Vaccine Injury Compensation Program (VICP): 1988–2019 [3], which highlighted VAPP risks as a key motivation for VICP establishment. KRI also published a commentary related to incentives for developing vaccine patches [4] and an economic analysis related to developing better defaults for valuing health outcomes based on health opportunity costs [5].

Prior to and during the COVID-19 pandemic, KRI published several studies related to updating the inputs of its global integrated model, including 2019 updates for iVDPV risks [160] (included in the prior review [1]). Following the release of the 2019–2023 GPEI strategic plan [161] and post-cessation strategy [162], KRI updated its integrated model inputs to use retrospective immunization and epidemiology data through the end of 2018 and prospective inputs consistent with available information about recent programmatic performance and updated plans. The third special issue of the journal *Risk Analysis* on Global Poliovirus Risk Management and Modeling, published in February 2021 [6], included many of these studies, several of which appeared online in 2020. In January 2020 (prior to the COVID-19 pandemic), the KRI update of its integrated model reported the GPEI was off-track with respect to achieving its polio eradication goals [161] and attributed this primarily to the low quality of OPV SIAs [7]. For this integrated model update [7], KRI reflected on key differences between the optimistic and ideal risk management assumptions it used prior to 2016 [163,164] and actual performance and plans after 2016 [8]. Due to its expectation of ongoing transmission of WPV1 in Pakistan and Afghanistan [7], KRI built on its prior Pakistan and Afghanistan modeling [165,166,167,168] to update its DEB model for these countries (as a single epidemiological reservoir) and identified pSIA strategies that could end WPV1 transmission within the timeline of the 2019–2023 GPEI strategic plan [9]. KRI also developed a DEB model of poliovirus transmission for northeast Nigeria to characterize the transmission of WPV1 that occurred in Borno and Yobe in 2016 [10]. Using this DEB as the basis for an SC model, KRI characterized the confidence of no circulation or undetected WPV1 transmission as a function of time for Borno and Yobe [11], which the Africa Regional Commission for the Certification of Poliomyelitis Eradication used to support its regional certification in 2020 [169]. KRI also updated its characterization of post-OPV cessation risks related to the misuse or inadvertent use of OPV after homotypic cessation, and updated its probability estimates for the need to restart OPV2 production and use in preventive immunization [12]. To support integrated analyses, KRI published updated cost estimates for poliovirus immunization and valuations of polio health outcomes [13], including consideration of health opportunity costs [5]. With global certification of WPV3 eradication in 2019 [170] and reductions of bOPV pSIAs, in late 2020, KRI published a DEB modeling study of the expected reductions of VAPP and cVDPV cases and vaccine dose requirements for a policy of globally coordinated cessation of type 3 OPV (OPV3) before type 1 OPV (OPV1) compared to ongoing bOPV use [14]. 

Following the May 2020 GPEI publication of an addendum to its 2019–2023 strategic plan that emphasized the use of nOPV2 for oSIAs [171], KRI applied its global model to explore the impacts of using nOPV2 compared to Sabin mOPV2 for oSIAs and reported that even with ideal characteristics, nOPV2 would not likely end all type 2 outbreaks and transmissions [15], which it also discussed in a commentary [16]. Considering pre-COVID-19 conditions, KRI updated its 2010 integrated analysis of the health and economic benefits of the GPEI [172] based on the status of the GPEI and its plans as of 2019 [17], which included consideration of the possibility of ending WPV1 transmission in Pakistan and Afghanistan [9]. KRI also applied its integrated model to consider the health and economic impacts of IPV or tOPV in RI with or without OPV use for oSIAs for different control and eradication scenarios for 2019–2029 [18]. 

After the emergence of COVID-19, a high-profile suggestion to use OPV to prevent COVID-19 in the United States [173,174] motivated KRI to perform a health economic analysis that estimated VAPP risks and costs with no anticipation of expected benefits for reducing COVID-19 [19] (and associated letter [175]). KRI explored the impact of disruptions caused by the COVID-19 pandemic on global polio eradication in a 2021 study [20], which included updating its assumptions related to long-range exportations. In 2021, KRI published two studies related to the hypothetical emergence of a novel poliovirus in 2020 to simulate a pandemic with poliovirus (instead of COVID-19) and demonstrated the consequences of policy decisions for control or eradication using nonpharmaceutical interventions [21] and vaccines [22]. KRI also updated its characterization of poliovirus transmission in Pakistan and Afghanistan post-COVID-19 to explore the impacts of different vaccine options for oSIAs (e.g., mOPV2, tOPV) in the context of the cocirculation of WPV1 and cVDPV2 [23]. A separate KRI study estimated the expected costs of poliovirus surveillance and immunization campaign quality monitoring for Pakistan and Afghanistan for 2019–2023 [24].

Outbreak response emerged as a theme for several KRI publications during this period. In 2021, KRI characterized the trade-offs of different OPV2 vaccines (i.e., mOPV2 or nOPV2) for oSIAs and the consequences of delaying oSIAs to wait for nOPV2 instead of using available mOPV2 [25]. This analysis emphasized the importance of responding quickly and well with available vaccine supplies (i.e., mOPV2) and anticipated that delays in waiting for nOPV2 would likely lead to over a thousand paralytic cases and greater global cVDPV2 transmission [25]. Despite this analysis, countries in the WHO African region chose to delay oSIAs to wait for nOPV2, which increased the number and spread of reported cVDPV2 cases and motivated KRI to ask, “What kind of world do we want?” in a commentary [26]. A separate commentary raised questions about the effectiveness of a nOPV2 for oSIAs and highlighted the increasingly complicated polio endgame [27].

Following up on discussions of secondary vaccine effects of OPV raised prior to the development of COVID-19 vaccines [19], KRI published a systematic review of secondary health effects of vaccines and policy insights for health economic analyses [28] and separately discussed the challenges of assessing the benefits and costs of non-polio and shared activities for polio and non-polio interventions in polio health economic analyses [29]. 

In 2022, following the publication of a new GPEI strategic plan for 2022–2026 [176], KRI published 2 analyses related to questions asked by GPEI partners during the development of the plan about the overall cases of polio prevented by poliovirus vaccines and GPEI [30] and the health and economic consequences of a shift to control in 2022 [31] (as an update to a 2007 KRI analysis of control vs. eradication [177]). Although KRI performed these studies in response to specific questions raised by GPEI partners, GPEI did not subsequently refer to the results of these studies. 

With new milestones [104] established in the new plan [176], KRI again updated its DEB model for Pakistan and Afghanistan [32]. Assuming the application of a pSIA strategy that KRI identified as successful for ending WPV1 transmission in its model [32], KRI used this analysis as the basis for an SC model to characterize the confidence of no undetected WPV1 transmission as a function of time [33]. The Global Commission for Certification of Poliomyelitis Eradication (GCC) used these results [32,33] to support a potential shortening of the time required to certify the end of indigenous WPV1 transmission after the last reported case or positive environmental sample to less than 3 years, depending on the quality of the surveillance and immunization information [178].

In 2022, KRI removed its assumptions related to OPV restart as a policy option in its global integrated model for consistency with observed experience. This change implied the potential use of OPV vaccines available for oSIAs for the full model time horizon of prospective models, and ended KRI characterization of the probability of OPV restart as a metric to characterize OPV cessation failure [34]. One application of the KRI global model published in late 2022 showed the trade-offs of different OPV2 vaccine options and oSIA characteristics (i.e., scope, timeliness, quality) [34], which identified aggressive options that could potentially lead to a high probability of cVDPV2 transmission dying out by the end of the model time horizon and characterized the low chances of success for the *status quo* GPEI strategy and policies. A separate study applied a DEB model of a hypothetical population with cocirculation of types 1 and 2 and demonstrated the advantages of coadministration of both types (e.g., tOPV or bOPV and nOPV2) compared to sequential or alternating strategies [35]. KRI also performed a look-back analysis to learn from its prior prospective modeling of oSIAs for managing OPV2 cessation [36].

In 2023, KRI explored the complexity of modeling the increase in vaccine options (i.e., nOPVs, different IPV formulations) and the dynamics of starting and/or stopping OPV in national immunization programs after OPV cessation [37]. KRI commented on polio eradication hurdles [38] and the need for updating expectations for nOPV2 [39]. KRI also reviewed the experience with the OPV2 stockpiles and discussed the challenges of forecasting vaccine demand given uncertainty about prospective national, regional, and global policies [40]. With ongoing discussions about potential global coordination of bOPV cessation, KRI also applied its global integrated model to characterize the expected outcomes of coordinated global cessation of bOPV use in 2027 without bOPV pSIAs [41], as suggested by the 2022–2026 strategic plan [176]. In addition to exploring the expected annual paralytic cases by type over time, KRI also explored the worst-case scenarios [42]. In the context of low expectations of successful bOPV cessation, a related health economic analysis anticipated a relatively low value of antiviral drugs [43].

Finally, in 2023–2024, KRI published a DEB model of the cVDPV2 transmission and IPV oSIA for the imported cVDPV2 outbreak that occurred in 2022 in New York State [44]. KRI used this as the basis for an SC model analysis that explored confidence about no circulation as a function of time since the last positive surveillance signal [45], which helped to support the New York State and national leaders feel more confident about the end of the outbreak. KRI also explored the trade-offs of different poliovirus vaccine options for outbreak response in IPV-using countries, such as the US 2022 cVDPV2 outbreak, and considered a hypothetical cVDPV1 outbreak to show differences between poliovirus types [46], which supported the 2023 US decisions to offer IPV to adults [179] and deliberations by the Advisory Committee on Immunization Practices (ACIP) about the potential use of OPV in the US for oSIAs at its February 2024 meeting. This study also included a systematic review of modeling studies that included IPV use in outbreak response [46].

#### 3.1.2. IC

The studies identified for IC for 2020–2024.25 showed its continued focus on statistical analyses of existing surveillance data and data collected as part of prospective trials or challenge studies and included a new focus area related to the development of a poliovirus direct detection method. With respect to transmission modeling, IC applied an SC model to characterize the spread of serotype-2 vaccine-derived-poliovirus outbreaks in Pakistan and Afghanistan to inform outbreak control strategies in the context of the COVID-19 pandemic [47], which built on a statistical analysis that quantified movement patterns and vaccination status in high-risk mobile populations [48]. 

Statistical analyses published by IC included an analysis of immune predictors of OPV immunogenicity among infants in South India [49]. One IC study used poliovirus genetic sequences from GenBank and demonstrated the application of phylogenetic and phylogeospatial modeling to infer geospatial transmission patterns of poliovirus transmission during the 2010 Tajikistan outbreak [50]. Another analysis characterized the variability in the sensitivity of environmental surveillance sites in Nigeria to detect poliovirus and other enteroviruses [51]. Two IC studies focused on risk factors for cVDPV2 transmission in Africa after 2016 [52,53]. The first of these highlighted the need for larger and faster OPV2 oSIAs to stop cVDPV2 transmission [52], and the second identified risk factors for VDPV2 emergence and suggested priorities for nOPV2 use [53]. IC reported the results of several vaccine effectiveness studies, including a phase 4 clinical trial on the effect of maternal immunization with multivalent vaccines containing IPV in infants [54] and the effectiveness of nOPV2 and mOPV2 against cVDPV2s in Nigeria between 2017 and 2022 [55]. IC commented on the first Africa-based nOPV2 clinical trial [56] and contributed to perspectives related to the impacts of one billion doses and WHO prequalification of nOPV2 [57] and the use of nOPV2 for oSIAs [58].

IC invested substantial effort in the research, development, and implementation of direct detection with nanopore sequencing (DDNS) for poliovirus surveillance [59,60,61,62]. This included publications that characterized rapid and sensitive direct detection and identification of poliovirus from stool and environmental surveillance samples using nanopore sequencing [59] and an analysis of the time taken to detect and respond to polio outbreaks in Africa considering the potential impact of direct molecular detection and nanopore sequencing [60]. IC also published a comparison of eleven RNA extraction methods for direct molecular detection of polioviruses from stool [61] and a prospective validation study of DDNS that reported preliminary cost estimates [62]. IC also included poliovirus surveillance in a consortium effort that it led with the goal of defining a research agenda for broad environmental wastewater surveillance of pathogens [63].

Following the detection of imported cVDPV2 transmission in the UK, IC published a commentary about the detection as a wake-up call [64] and a statistical analysis of the sustained detection by enhanced environmental surveillance in London sewage between February and July 2022 [65]. IC contributed substantially (i.e., three of eight coauthors) to a review of the use of IPV for oSIAs [66] prepared at the request of the SPWG for discussion at its 24th meeting (August 2022) and the October 2022 SAGE meeting.

#### 3.1.3. LSHTM

Our review identified one SC modeling study that explored the effect of population partitioning on the probability of silent poliovirus transmission, which included a coauthor affiliated with both LSHTM and SACEMA [67]. The lead author of this study [67] published several poliovirus transmission modeling studies counted as “other transmission studies” in the prior review [1], in collaboration with a polio modeler from the University of Michigan (Dr. James Koopman). This study [67] represented the only publication that met the review inclusion criteria for SACEMA, although for this review, we attributed it to LSHTM.

As discussed earlier, several IC studies in the prior review included a researcher who moved to LSHTM in 2018 [1]. Not surprisingly, given prior collaboration, several LSHTM-led studies published in 2020–2024.25 included coauthors from IC [68,69,70]. One study included a coauthor from IDM/BMGF [71]. LSHTM reported statistical epidemiological analyses that characterized changes in the epidemiology of poliovirus serotype 2 following OPV2 cessation [68], which followed discussions held during a January 2020 meeting that included all of the groups. Other LSHTM studies explored the optimization of environmental surveillance to detect poliovirus importations into England and Wales [69] and characterized the epidemiology of cVDPV2 global outbreaks between 2016 and 2020 [70]. Another statistical analysis characterized the impact of surveillance and other factors on the detection of emergent and circulating vaccine-derived polioviruses [71]. We identified several LSHTM reviews related to the polio endgame [72], the accelerated roll-out of the nOPV2 vaccine [73], and challenges with achieving polio eradication given population immunity as of early 2024 [74]. LSHTM coauthors also commented on the challenges of informative wastewater sampling for SARS-CoV-2, considering lessons learned from polio eradication [75]. LSHTM also published a vaccine effectiveness study of an mOPV2 challenge dose given to IPV-vaccinated children [76], a mucosal immunity study for nOPV2 in healthy adults [77], and a review of polio mucosal immunity studies [78].

#### 3.1.4. IDM/BMGF

IDM/BMGF published three poliovirus transmission modeling related studies between 2020 and 2024.25 [79,80,81]. Using an IB model, the first study incorporated the results from a mOPV2 clinical trial performed in Bangladesh, which suggested that household and community structure played an important role in limiting transmission [79]. In 2023, the second study described the development of a DEB model that endogenously included OPV2 reversion for the same population [80]. The third study applied a DEB model to revisit the role of time-varying viral shedding in modeling environmental surveillance using the 2013 poliovirus outbreak in Israel [81]. 

IDM/BMGF also published multiple statistical analyses. One study modeled the genetic sequences of type 2 polioviruses and identified positive selection and tight transmission bottlenecks that substantially influenced the early evolution of OPV2 [82]. Another study of disease surveillance investments and administrative data for Pakistan showed the limited value of the available information for improving surveillance quality [83]. Studies in 2023 developed a real-time prediction model of cVDPV2 outbreaks to aid oSIA decisions [84] and analyzed changes in population immunity for all three types of polioviruses since the 2016 shift from tOPV to bOPV for selected countries and regions [85]. The final IDM/BMGF publication applied a time series statistical model to estimate the vaccine effectiveness of nOPV2 in oSIAs in Nigeria, which also reported similar vaccine effectiveness of mOPV2, but substantially lower effectiveness for IPV than for either OPV2 [86].

#### 3.1.5. GIT

GIT published its first two poliovirus transmission modeling studies in 2024, including a peer-reviewed conference publication [87] and a related journal article [88]. Both studies described the application of a DEB model it developed to characterize cVDPV2 transmission and the impacts of interventions that aim to control outbreaks in Nigeria, and they identified the need for more aggressive oSIAs (i.e., more rounds and broader coverage, particularly in under-vaccinated communities) [87,88].

#### 3.1.6. Economic Analyses Published by Other Groups

The search identified two other studies published in 2020 that presented game theoretic applications to polio, which we included as other economic analyses [89,90]. The first integrated a DEB model with two age classes (i.e., [180] identified in the prior review [1]) into the game theoretic model, which led us to characterize it as an integrated model [89]. The study concluded that achieving polio eradication would require a mandatory vaccination policy [89]. The second study used survey data related to perceptions of the population benefits of OPV and statistical model inputs informed by poliovirus transmission characteristics in a model that suggested that prosocial behavior to receive bOPV in Israel contributed some to the oSIA vaccine uptake after the 2013 WPV1 outbreak [90].

The search found three other polio-related economic modeling studies. These include cost-effectiveness analyses of three poliovirus immunization schedules in Shanghai, China [91] and various immunization schedules with Sabin IPV in Hangzhou, China [92]. The final study assumed OPV could provide protection from COVID-19 due to secondary effects and suggested the need to prioritize clinical trials and other studies that could resolve the uncertainty about the effects of OPV on COVID and other non-poliovirus diseases [93].

#### 3.1.7. Poliovirus Transmission Modeling Studies Published by Other Groups

Our review identified nine other studies that presented DEB models of poliovirus transmission for theoretical populations using different numerical simulation methods [94,95,96,97,98,99,100,101,102].

### 3.2. Trends in Characteristics of Polio Modeling Studies

Table 1 provides a high-level comparison between the numbers and types of studies included in this review compared to those in the prior review for 2000–2019 [1] (with the updates discussed above). Notably, the 102 studies identified for the 4.25 years covered in this review imply an increase in the annual rate of polio modeling papers published over time of approximately 2, 5, 9, 22, and 24 papers/year, respectively, for 2000–2004, 2005–2009, 2010–2014, 2015–2019, and 2020–2024.25. Among the modeling groups, KRI published the largest number of studies for 2020–2024.25 (46 publications), followed by IC (20 publications), and LSHTM (12 publications). 

Table 2 summarizes the themes explored in the included studies by all groups with publications discussed in the prior section. We identified publications by multiple groups for several key themes from the prior review [1], as well as several new themes. Given the global situation with ongoing poliovirus transmission, particularly for cVDPV2s, we identified publications by all the groups in Table 2 on: outbreak response speed (and quality), population immunity, and novel OPV, particularly nOPV2. All groups discussed changes in population immunity in multiple publications, albeit with different definitions. As in the prior review [1], outbreak response speed and quality represented a major theme, with all groups demonstrating the need for improvements in oSIAs. With the use of nOPV2 in oSIAs starting in 2021, our review identified publications by all groups related to nOPV2, many of which included analyses related to nOPV2 use in oSIAs. With respect to OPV cessation dynamics, several groups explored the evolution of OPV, which included some prospective modeling related to potential bOPV cessation. 

Also represented in Table 2 as related to OPV cessation dynamics, we noted multiple studies by different groups on the failure of the 2016 globally coordinated cessation of OPV2 use for preventive immunization, which included retrospective statistical analyses of epidemiological data and poliovirus transmission modeling. With substantial GPEI investments in the expansion of environmental surveillance, Table 2 also shows numerous studies by different groups related to surveillance. Notably, given multiple publications by IC related to its development of DDNS, we broadened the category from environmental surveillance to surveillance.

Most of the modeling groups also published on the topic of IPV use. These publications included IPV use for oSIAs, with some studies motivated by actual experiences (e.g., the 2022 cVDPV2 outbreak in the United States). In addition, multiple groups published studies related to the role of IPV use after OPV cessation, but we did not include the studies that simply added IPV to RI consistent with the prerequisites for OPV2 cessation in Table 2. Not surprisingly, most modeling groups published studies motivated by the global experience with the COVID-19 pandemic, including many studies related to disruptions in national and GPEI immunization activities. Two groups published studies related to undetected circulation in different geographies. Only KRI published studies related to the topics of expanded age group SIAs, silent transmission on an IPV background and/or delayed detection of transmission due to IPV use, vaccine stockpile, iVDPVs, containment, secondary effects of OPV, and GPEI transition and integration. 

The bottom portion of Table 2 shows the geographic scope covered by 45 studies that included poliovirus transmission modeling [7,9,10,11,12,14,15,17,18,19,20,21,22,23,25,30,31,32,33,34,35,36,41,42,43,44,45,46,47,67,79,80,81,87,88,89,94,95,96,97,98,99,100,101,102]. Not surprisingly, two groups published studies on Pakistan, Afghanistan and Nigeria, which continue to represent critical geographies for polio eradication. Populations modeled by only one group include Bangladesh, Israel, and the United States, and only KRI performed global analyses.

### 3.3. Modeling in GPEI Annual Reports or SAGE Conclusions and Recommendations

Table 3 provides excerpts of references to modeling and/or modeling groups and the sources for results reported that we identified in the GPEI Annual Reports. These discussions of modeling activities and/or references to studies and results published by the modeling groups provide some indication that GPEI broadly relied on modeling inputs. The rightmost two columns show the modeling group(s) referred to by name or by the journal of their publication, and the references to studies from which the GPEI Annual Reports included mention of specific results. Between 2005 and 2018, the GPEI Annual Reports referred to results from studies published by 4 modeling groups: LSHTM [121], KRI [172,177,181,182,183], IC [184,185,186,187], and IDM [188], and we noted multiple references to the results from older studies over time. References to the results from modeling studies occurred in 4 of 5 years for the 2005–2009, 2010–2014, and 2015–2019 time periods. In the 2020–2022 GPEI Annual Reports, only the 2022 report even alluded to the results from modeling studies by including a link to a 2022 investment case [189] that presented the results of an unpublished update of a prior study [188] and mentioned the outdated results of a 2007 publication [177]. Notably, the investment case did not mention a published 2021 updated economic analysis of the GPEI [17]. Two other relevant studies published shortly after publication of the investment case included quantification of the cases of polio prevented by polio vaccines and GPEI [30] and an update of the consequences of shifting to control in 2022 [31] (as an update for an outdated study of control vs. eradication [177]).

Table 4 provides excerpts of discussions of polio modeling (and economic analyses) we identified in SAGE meeting conclusions and recommendations published in the *Weekly Epidemiological Record* [132]. Although every regular SAGE meeting agenda included polio eradication (annually 2000–2002 and biannually 2003–2024), Table 4 only includes meetings for which we identified discussions of polio modeling results. The third column provides references to publications specifically discussed as studies or reviews, as SAGE noted in the conclusions and recommendations. The fourth column provides references to publications we could identify in our review of available presentations to SAGE. Although we identified multiple references to modeling and/or the modeling groups, we identified only five explicit references to specific studies or reviews published by the modeling groups (i.e., KRI [172,177], IC [66,184,185]) and one acknowledgment of IC unpublished work supporting WHO. However, our review of the available presentations made to SAGE identified the inclusion of results from the modeling groups in most of its meetings. We recognized that some of the SAGE meetings included discussions of topics for which relevant modeling available at the time did not get presented (e.g., the role of older age groups in transmission, the economics of using IPV in outbreak response, trade-offs between vaccine choices for outbreak response, including in areas with cocirculation of poliovirus types 1 and 2). We identified some unpublished and not peer-reviewed (to date) modeling results presented (see notes at the bottom of Table 4), most notably predictions about the number of cVDPV2 outbreaks to expect after OPV2 cessation that did not imply any dependence on the implementation of oSIAs. We also observed multiple references to both ACPE and the SPWG. This motivated us to also review the available notes for the record from the face-to-face meetings of these groups. We know from experience that modeling groups attended and presented at some ACPE meetings, but we did not find agendas for all ACPE meetings, and we did not find specific references to the modeling publications presented in the ACPE meeting reports. 

Table 5 shows the representation of the modeling groups in the 27 face-to-face SPWG meetings and the dates of the meetings. The third through sixth columns show the roles for four of the modeling groups (i.e., KRI, IC, LSHTM, and IDM/BMGF) by indicating the meetings for which representatives from the group participated as a member (M), an invited external participant (P), or a rapporteur (R). The SPWG generally holds closed meetings, with participation limited to committee members and invited participants, although some representatives of some GPEI core partners also attended SPWG meetings. Representatives of the modeling groups, attended different numbers of total SPWG meetings to date: KRI (14), IC (22), LSHTM (12), and IDM/BMGF (24). As shown in Table 5, which uses an asterisk (*) to indicate a presentation, members frequently presented work from their groups and nearly all invited external participants presented work from their group while at the meeting. The seventh column shows the topic presented by modeling groups (and indicates the group). We identified some presentations that included work from modeling groups not attending the meeting. We included the topic of the presentation and added a note to indicate these presentations. This led to the inclusion of the topics of some presentations in the seventh column with no group indicated, as well as some notes showing presentations given by a group representative that included work from one or more other modeling groups in addition to their own work. As shown in the rightmost column in Table 5, between 2008 and 2014 the SPWG reports included references to specific studies by different modeling groups. However, since 2015, while some SPWG notes for the record mentioned presentations by modeling groups, they did not include references to specific studies (e.g., the IC review of IPV use for oSIAs [66]). In the absence of reviewing each presentation, we could not identify all of the modeling results presented to the SPWG.

Overall, it appears that poliovirus transmission and economic modeling results played a much larger role in GPEI and SAGE discussions in earlier time periods than during 2020–2024.25, although we see a strong representation of consideration of the results of the modeling groups for 2000–2024.

## 4. Discussion

Publications by the modeling groups since 2020 have continued to show the application of different approaches and active communication of model results to policymakers. However, we observed fewer references to modeling results in GPEI Annual Reports since 2020 than during the prior 15 years, and a decrease in the impact of the results of poliovirus transmission and economic modeling on decisions. Notably, in some cases, the reduced impact of modeling occurred despite the agreement of the multiple modeling groups. For example, although multiple modeling groups recommended a rapid response to cVDPV2 outbreaks using any available OPV2 vaccine (including mOPV2) and SAGE endorsed this recommendation, some countries in the African region delayed their responses to cVDPV2 outbreaks after 2020 to wait for nOPV2 because of its perceived higher safety profile. Multiple modeling groups also published studies that highlighted the late, small, and low quality of oSIAs in some areas as a primary reason for OPV2 cessation failure.

During the past 5 years, modeling studies suggested an expected reduction in overall incremental net benefits of the global polio eradication efforts, decreased chances of a successful polio eradication endgame, expectations of increased costs of polio immunization prospectively due to the shift from OPV to IPV in RI associated with the increase in recommended IPV doses in RI, and the complexity of continued use of different formulations of OPV. However, commitment to “finishing the job” remains strong, in spite of a less favorable economic impact. Consistent with this, discussions by GPEI and SAGE trended more toward a focus on polio epidemiological studies, equity (particularly gender equity [154]), and integration of GPEI activities into national immunization programs. The increased focus on statistical analyses of polio epidemiological data reflects the reality of the increased incidence of polio cases reported since 2020 [104]. 

Similar to the prior review [1], we looked for areas of disagreement about recommendations from the modeling groups. We could not formally document some known differences from internal modeling group discussions because they involved unpublished results. For example, published modeling emphasized the importance of rapidly responding to cVDPV2 outbreaks using the available mOPV2 given the limited availability of nOPV2 [25,34], which unpublished modeling by one other group supported and unpublished modeling by another group did not support (it suggested that countries should wait to use nOPV2 before using mOPV2 in oSIAs). In addition, the communications to countries related to the roll out and promotion of nOPV2 in 2021 promised a better vaccine than mOPV2, which effectively disincentivized the use of mOPV2, devalued the available mOPV2 stockpile, and created challenges with respect to using any Sabin OPV in SIAs to increase and maintain population immunity. 

With respect to Pakistan and Afghanistan, modeling performed by KRI, IC, and IDM did not lead to the accelerated achievement of WPV1 eradication in Pakistan and Afghanistan. We could not assess the agreement between these since some of the analyses remain unpublished, but studies by KRI and IC agreed with the need to quickly restart immunization in Pakistan and Afghanistan following disruptions caused by COVID-19 [32,47]. In addition, insights from modeling did not succeed in preventing the failure of OPV2 cessation, despite repeated warnings of its likely failure as early as 2017 in Cessation Risk Task Team meetings that occurred between June 2016 and April 2021, which included all of the modeling groups along with representatives from most GPEI core partners. Modeling support of stockpile needs prior to OPV2 cessation did not account for shifts in GPEI leadership and governance and the resulting adoption of lower prioritization of SIAs [104], decentralization of decision making, or the apparent willingness to accept increased polio cases to wait for a different vaccine (i.e., nOPV2). Notably, prior to OPV2 cessation, prospective modeling assumed aggressive oSIAs with mOPV2 would either successfully stop all type 2 poliovirus transmission or countries would restart the use of tOPV in RI [36], and thus developed estimates of demand for a time-limited OPV stockpile [40]. Instead, global efforts moved toward increased use of IPV, the development and deployment of new vaccine tools, including nOPV, and the associated extension of the polio endgame. Given the current epidemiological situation, we can anticipate that future publications of polio modeling studies may support another update of the systematic review. 

Limitations of this review include our inability to evaluate whether and how national and regional decision makers interact with each other, GPEI, and other global health policy makers. Although we described some work by the modeling teams used by national and regional decision makers (e.g., New York modeling, the African Regional Certification Commission), these represent anecdotal examples, not the results of a systematic process to collect information from decision makers at all levels about their use of modeling results. Modeling results represent only one of the factors that may influence decisions.

## 5. Conclusions

The representatives from some modeling groups continued to serve as members on the SPWG and the modeling groups continue to increase and to publish their work at an increasing rate. By combining and updating results from prior review [1], for the full time period of 2000–2024.25 we identified 246 polio publications by the modeling groups KRI (124), IC (64), LSHTM (29), IDM/BMGF (27), and GIT (2), plus 46 publications by others (including 32 poliovirus transmission modeling papers and 14 economic analyses). With the initial target for achieving global polio eradication in 2000, the increasing number of modeling groups and rate of publications contrast notably with expectations of successful eradication and dissolution of the GPEI long before 2024. In spite of this, the GPEI core partners remain committed to delivering on the promise of polio eradication, and this suggests that opportunities and support for polio modeling will continue for the foreseeable future.

## Figures and Tables

**Table 1:** Characteristics of included studies with comparison to results reported for 2000–2019 [1].

Time Period	2000–2019 [1]	2020–2024.25	
Modeling group (first publication year) KRI (2003) IC (2006) LSHTM (2000) * IDM/BMGF (2014/2021) * SACEMA (2022) * GIT (2024) * Other economic analyses (2000) Other transmission modeling (2000)	Count (number excluded) 78 44 ^a,b^ (1 ^c^) 17 ^c^ (1 ^b^) 19 (2 ^a^) NA NA 9 23	Count (number excluded) [References] 46 [1,2,3,4,5,6,7,8,9,10,11,12,13,14,15,16,17,18,19,20,21,22,23,24,25,26,27,28,29,30,31,32,33,34,35,36,37,38,39,40,41,42,43,44,45,46] 20 (3 ^d^) [47,48,49,50,51,52,53,54,55,56,57,58,59,60,61,62,63,64,65,66] 12 ^d,e,f^ [67,68,69,70,71,72,73,74,75,76,77,78] 8 (1 ^e^) [79,80,81,82,83,84,85,86] 0 (1 ^f^) [67] 2 [87,88] 5 [89,90,91,92,93] 9 [94,95,96,97,98,99,100,101,102]	
Publication year 2000–2004 2005–2009 2010–2014 2015–2019 2020–2024.25	Count {Rate per year} 10 {2} 26 {5} 45 {9} 109 {22}	Count {Rate per year} 102 {24}	
Study type Integrated (DEB and economic) DEB SC IB DES, DEB DEB, SC DEB, IC Economic/cost analysis only ** Risk assessment ** Vaccine effectiveness ** Mucosal immunity ** Reviews ** Discussions of policy options ** Perspectives/commentaries **	Count 12 42 14 9 3 2 1 15 20 17 6 14 5 30	Count 6 ^g^ 34 4 1 0 0 0 10 18 3 3 8 1 14	

Abbreviations: BMGF, Bill and Melinda Gates Foundation; DEB, differential-equation-based model; DES, discrete-event simulation model; GIT, Georgia Institute of Technology; GPEI, Global Polio Eradication Initiative; IB, individual-based model; IC, Imperial College; IDM, Institute for Disease Modeling; KRI, Kid Risk, Inc.; LSHTM, London School of Hygiene and Tropical Medicine; SC, stochastic compartmental; SACEMA, South African Centre for Epidemiological Modelling and Analysis.

* Only KRI, IC, IDM included in the prior review as GPEI-partner-supported modeling teams covered under the GPEI Data Sharing Agreement as of 2019, although LSHTM researchers published studies noted in the prior review [1]. Other modeling groups added (including LSHTM) reflect the expansion of the GPEI Data Sharing Agreement. BMGF absorbed IDM in 2021, such that we continued to include IDM/BMGF studies published by IDM authors in the prior review [1] after 2021 as IDM/BMGF (see text).

** Categories only applicable to studies published by KRI, IC, IDM/BMGF, LSHTM, SACEMA, and GIT.

^a^ Two IC studies included an IDM coauthor [1].

^b^ One IC study from the prior review included an LSHTM coauthor [1].

^c^ LSHTM papers published 2000–2019 are now reclassified (see Methods), including 2 papers with IC coauthors [1] now counted here [111,112], one DEB modeling study published in 2001 [113] moved from the other transmission models category [1], one statistical epidemiological study (Risk assessment) [114], four mucosal immunology studies [115,116,117,118], nine perspectives/commentaries [119,120,121,122,123,124,125,126,127].

^d^ Three LSHTM studies included an IC coauthor [68,69,70].

^e^ One LSHTM study included an IDM coauthor [71].

^f^ One LSHTM paper included a coauthor with both an LSHTM and SACEMA affiliation [67].

^g^ One game theoretic analysis is counted here as an integrated model instead of an economic or cost model due to its inclusion of a dynamic transmission model [89].

**Table 2:** Summary of themes explored and specific populations in poliovirus transmission model studies by group.

Modeling Group	KRI	IC	LSHTM	IDM /BMGF	GIT	Other	
Theme							
Outbreak response speed/quality	[7,12,15,23,25,34,35,36,44,46]	[47,52,53,55,60,62]	[70]	[84,86]	[87,88]	[90]	
Expanded age group SIAs	[34]						
Population immunity*	[7,9,10,11,12,14,15,17,18,19,20,21,22,23,25,30,31,32,33,34,35,36,41,42,43,44,45,46]	[47,52,53]	[70,74]	[82,83,84,85,86]	[87,88]		
OPV cessation dynamics	[14,41,42]						
Silent transmission on an IPV	[44,45,46]	[52,53]	[68,70,71]	[80,85]			
background/delayed detection							
of transmission due to IPV use							
Role of IPV after OPV cessation	[17,18]	[54]				[91,92]	
Undetected circulation	[11,33,45]		[67,69]				
Role of IPV in oSIAs	[22,31,44,46]	[55,66]			[87,88]		
(Environmental) surveillance	[10,11,24,33,44,45]	[50,51,59,60,61,62,63,64,65]	[69,71,75]	[81,83]			
Vaccine stockpile	[40]						
iVDPVs	[43]						
Novel OPV (nOPV)	[13,15,16,20,22,23,25,26,27,34,35,36,37,38,39,40,41,42,43,46]	[55,56,57,58]	[72,73]	[86]	[87,88]		
COVID-19/pandemic modeling	[19,20,21,22]						
Secondary effects of OPV	[19,20,28,29]	[47]				[93]	
GPEI transition/integration	[29]						
Containment	[12]						
Geographic area modeled for studies with poliovirus transmission models							
Global	[7,12,14,15,17,20,21,22,25,30,31,34,36,41,42,43]						
Pakistan/Afghanistan	[9,19,23,32,33]	[47]					
Nigeria	[10,11]				[87,88]		
Bangladesh				[79,80]			
Israel							
United States	[18,44,45,46]						
Hypothetical	[35]		[67]			[89,94,95,96,97,98,99,100,101,102]	

Abbreviations: BMGF, Bill and Melinda Gates Foundation; GIT, Georgia Institute of Technology; GPEI, Global Polio Eradication Initiative; IC, Imperial College; IDM, Institute for Disease Modeling; IPV, inactivated poliovirus vaccine; iVDPV, immunodeficiency-associated vaccine-derived poliovirus; KRI, Kid Risk, Inc.; LSHTM, London School of Hygiene and Tropical Medicine; NA, not applicable; nOPV, novel OPV; OPV, oral poliovirus vaccine; oSIA, outbreak response SIA; SIA, supplemental immunization activity.

* Modeling groups use different definitions for population immunity [1].

**Table 3:** Discussions of work related to polio modeling and groups in GPEI Annual Reports [133,134,135,136,137,138,139,140,141,142,143,144,145,146,147,148,149,150,151,152,153,154,155,156].

Year	Excerpt	Group	Study
2000	Refers to plans to develop a “strategy for stopping vaccination that will evaluate a number of proposed strategies, including routine immunization with the inactivated polio vaccine (IPV) or an OPV ‘pulse’ immunization followed by cessation”		
2001	“WHO will develop policy decision models over the next 12 months that reflect how the range of possible research outcomes would affect post-certification policy development”		
2002	A “framework has now been developed that summarizes these risks into two major categories, including (a) those due to VDPV, and (b) those due to the handling of wild poliovirus stocks”		
2003	Key decisions in 2003: 1. Cessation of oral polio vaccine (OPV) 2. No universal introduction of the inactivated polio vaccine (IPV) 3. Strategies for the Safe Cessation of OPV “Guidelines for national decision-making on OPV cessation will be completed, outlining: (a) rationale for OPV cessation; (b) risks of polio in the post-OPV era; (c) surveillance requirements; (d) post-OPV stockpile and response; and (e) implications of IPV introduction”		
2004	Concluded that “eradicating all forms of poliomyelitis paralysis will require eventually stopping use of OPV globally and that the cessation of OPV must be implemented simultaneously across the world”		
2005	“A comprehensive approach must be taken to optimize the management of the risks of either the re-emergence of polio due to a cVDPV or the re-introduction of either a wild or Sabin poliovirus, following the global interruption of wild poliovirus transmission.” “The ACPE, in October 2005, issued new international standards for polio outbreak response to guide countries in planning and responding to any importations of virus” and “further expansion of mOPV use in supplementary immunization activities”, and “assessment was conducted on the consequences of a poliovirus release during or after OPV cessation (Fine P. E. M. and Ritchie S., Consequences of release/reintroduction of polioviruses in different geographic areas after OPV cessation. Risk Analysis, 2006)”	LSHTM, KRI	[121,181] ^a,b^
2006	“Research published in Science magazine indicates monovalent OPV can boost immunity enough to stop polio in northern India.” “The humanitarian and economic case for finishing eradication is sound. A new study from Harvard University demonstrates that over a 20-year period, controlling polio at high levels would cost more in human suffering and dollars than finishing eradication.”	IC, KRI ^a^	[177,184]
2007	“To examine the assumptions underpinning current planning for the mOPV stockpile, a Harvard University/Massachusetts Institute of Technology collaboration continues to conduct mathematical modeling of outbreak response activities for polioviruses following OPV cessation.” “In close collaboration with the Imperial College of London, studies were undertaken to better estimate the efficacy of mOPV 1 and 3 in different field settings (India, Nigeria, and Pakistan).”	KRI ^a^, IC	[182,185]
2008	“Studies showing increased efficacy of monovalent type 1 oral polio vaccine (mOPV1) over trivalent oral polio vaccine (tOPV) in Nigeria are published in the New England Journal of Medicine, affirming the feasibility of rapidly stopping polio in that country.” “In 2008, the PRC made a grant to Kid Risk Inc., formerly part of a Harvard University and Massachusetts Institute of Technology collaboration, to continue mathematical modeling of outbreak response scenarios for polioviruses following OPV cessation to further inform policy in this area.”	IC, KRI	[183,186]
2009	“…An extensive program of research was accelerated in 2009 to develop the tools and policies to minimize and manage the long-term risks of polio.” “To prepare for the management of long-term poliovirus risks, the GPEI is focusing research and policy development on three major areas: (1) better characterizing the primary long-term poliovirus risks (ie cVDPVs, VAPP, iVDPVs, and residual stocks of WPVs, VDPVs and Sabin viruses); (2) developing new products to manage the risks associated with OPV cessation, including the development of an international stockpile of mOPVs for cVDPV response and affordable IPV options for low-income countries that perceive the medium or long-term risks of poliovirus warrant continued routine immunization after OPV, cessation. (3) Establishing mechanisms to internationally coordinate risk management strategies, particularly the application of appropriate safeguards and bio-containment conditions for the handling and storage of residual polioviruses and potentially poliovirus-infected materials, the synchronization of the cessation of routine immunization with OPV and the adherence to internationally-agreed processes for the post-eradication use of OPV in response to new cVDPVs.”		
2010	“In a rigorous evaluation of the benefits and costs of eradicating polio, a study published in Vaccine finds that the program could provide net benefits of at least US$40–50 billion by 2035, mostly in low-income countries, if transmission of wild polioviruses is interrupted within the next five years.” “Jenkins et al, published in the New England Journal of Medicine in June 2010, analyzed the largest-ever recorded cVDPV outbreak detected in Nigeria.”	KRI, IC	[172,187]
2011	“Mathematical modeling shows that failure to eradicate the remaining 1% puts the world at significant risk of polio resurgence, potentially leading to over 200,000 children paralyzed annually within a decade.”		[177] ^c^
2012	“On one side of the balance, a lasting world free of polio where no child will ever know the pain of polio paralysis and US$ 50 billion in economic benefits; on the other, a resurgence of the disease resulting in 200,000 cases every year within 10 years. All countries will benefit equally from global success.”		[177] ^c^
2013	“Type 2 outbreak response principles were endorsed by the SAGE in November 2013. It was decided that outbreak response should utilize both monovalent OPV type 2 and IPV to rapidly boost and establish population immunity around the outbreak response zone to prevent the emergence of cVDPV. The use of mOPV2 is needed to induce intestinal immunity among those who have not been vaccinated against type-2 previously.”		
2014	“In 2006, the World Health Assembly issued international outbreak response guidelines with specific measures countries should take upon detection of a polio outbreak in any polio-free area. Full implementation of these guidelines reduced the extent (in time and number of cases) of new outbreaks by 50% compared to previous outbreaks. Outbreak response is now more critical than ever, as the world is now closer than ever to being polio-free and the phased removal of oral polio vaccines (OPV) is beginning. That is why the GPEI has issued revised international outbreak response guidelines to countries, building on those from 2006.”		^b^
2015	“A polio-free world will reap savings of more than US$ 50 billion, funds that can be used to address other pressing public health and development needs.”		[172] ^c^
2016	“A polio-free world will result in global savings of US$ 50 billion (mostly in developing countries).”		[172] ^c^
2017	“Failure to eradicate polio would result in a drastic resurgence of the disease globally, and within the next 10 years, the world could again see 200,000 new cases every single year.” “A world without polio will result in savings of more than US $50 million.” “"Globally, a polio-free world will reap savings of over US$50 billion, funds that can be used to address other pressing public health needs”. “Even if it has taken longer and cost more than all had anticipated, the goal of a polio-free world — so near at hand — is worth pursuing, for the benefit of all generations of children to come.” “In addition to the significant humanitarian benefits associated with polio eradication, the effort is also associated with substantial economic benefits. A world free of polio will result in savings of more than US$50 billion, which can be used to address other critical public health and development needs.”		[177] ^c^ [172] ^c^
2018	“Achieving a polio-free world will generate an estimated US$14 billion in cumulative cost savings by 2050, compared to the cost countries would incur to control the virus indefinitely. In financial terms, the global effort to eradicate polio has already saved more than US$ 27 billion in health costs since 1988.”		[188]
2019	“…The continued spread of existing cVDPV2 outbreaks and the emergence of new cVDPV2s pointed to the insufficient quality of outbreak response with monovalent oral polio vaccine type 2 (mOPV2). The risk that these strains spread further or that new strains emerge was magnified by an ever-increasing mucosal immunity gap to type 2 poliovirus on the continent, following the switch from trivalent oral polio vaccine (tOPV) to bivalent oral polio vaccine in 2016.”		
2020	“Analysis of data for 2020 shows an increase of 0 dose cases and a decrease in 3 plus doses case both in endemic and outbreak countries. This is probably a consequence of the COVID-19 pandemic and related disruptions of immunization campaigns.”		^d^
2021	“A statistical analysis of zero-dose children was performed in 2021 using 2016–2020 sex-disaggregated surveillance data for children aged 0–36 months. It was conducted through an intra-cluster correlation coefficient and adjusted multiple regression analysis to calculate risk at the province and district levels in endemic countries (Afghanistan and Pakistan). Age and sex were used as risk variables to calculate the risk of children being among those who do not receive any dose and to calculate if this risk was equally distributed across the countries. The results were mapped to showcase the districts in which children had a higher risk of not receiving any doses.”		^d^
2022	“The poliovirus is cornered to just a few high-risk geographies, but there is no room for complacency. Continuing to invest in polio eradication could save the world over US$ 30 billion in health care cost savings this century, compared to the cost of just controlling polio.”		^e^
2023	NA		

Abbreviations: ACPE, Advisory Committee on Polio Eradication; cVDPV, circulating VDPV; GPEI, Global Polio Eradication Initiative; IC, Imperial College; IPV, inactivated poliovirus vaccine; iVDPV, immunodeficiency-associated VDPV; KRI, Kid Risk, Inc.; LSHTM, London School of Hygiene and Tropical Medicine; mOPV, monovalent OPV; NA, not available; OPV, oral poliovirus vaccine; OPV#, type # OPV (# = 1, 2, or 3); PRC, Polio Research Committee (of the GPEI); SAGE, Strategic Advisory Group of Experts on Immunization for the World Health Organization; tOPV, trivalent OPV; VAPP, vaccine-associated paralytic polio; VDPV, vaccine-derived poliovirus; WHO, World Health Organization; WPV, wild poliovirus.

^a^ Kid Risk, Inc. started as the Harvard Kids Risk Project with affiliation with the Massachusetts Institute of Technology in 2004–2007.

^b^ In 2005, Kid Risk, Inc. presented modeling to ACPE [190] related to improving outbreak response [181,191] that supported the 2006 outbreak response guidelines. In 2005, ACPE also heard presentations about other modeling studies published in a 2006 special issue of Risk Analysis led by KRI [192], including the cited LSHTM study [121] and another cited paper on containment [193].

^c^ Results from an older study mentioned in one or more prior annual reports (see above).

^d^ No source for statistical analysis was mentioned. ^e^ Links to the 2022 GPEI Investment Case [189], which refers to an unpublished update of [188] and outdated results reported in [177].

**Table 4:** Polio modeling discussions identified in SAGE meeting conclusions and recommendations.

Meeting Date	Excerpt	Study/Review	Presented at the Meeting
November-1999	“A delay in achieving the polio eradication target would increase the cost of the initiative by as much as US$ 100 million per year. In addition, it would be very difficult to sustain current funding levels for more than 24–36 months, a crucial point for those polio-free countries that would need to continue holding national immunization days beyond the target date to assure freedom from wild poliovirus”		
June-2002	“SAGE reaffirms the importance of the substantial programme of work now devoted to the development of polio immunization policy for the post-certification era. The immediate focus of this policy work should be in evaluating the feasibility of eventually stopping the routine use of oral polio-vaccine (OPV) worldwide” “SAGE recommends that the framework be supplemented by: [1] a peer review of the estimated burden of VAPP, cVDPV, and iVDPV; [2] a geopolitical/cultural understanding of how the ‘perceived risks’ that can not be answered by the scientific research agenda alone (e.g., bioterrorism, VAPP) may affect the post-certification policy in OPV-using countries; [3] an economic assessment of the various options; and [4] the completion of the research agenda to better define the risks in the post-certification era and the strengths and weaknesses of the risk management strategies”		
June-2004	“SAGE recommended that, to assist its deliberations on post-OPV immunization policy, WHO should keep it fully informed of: all related policy decisions made by the oversight groups responsible for other aspects of the OPV cessation work (i.e. the Ad-hoc Advisory Committee on Polio Eradication, the Global Commission on Certification of Polio Eradication, the Biosafety Advisory Group); the evolving understanding of the nature and magnitude of the risks of circulating polioviruses following interruption of wild poliovirus transmission and OPV cessation; and the outcomes of the continuing work to model these risks over time.”		
November-2005	“SAGE also applauded the work in progress on the post-eradication strategies”		
April-2006	“SAGE noted the ratification by ACPE of the new international standards for outbreak response”		
April-2007	“An independent analysis was presented to SAGE that supported the humanitarian and economic case for investing heavily to finish eradication. The study, published in the Lancet, showed that polio “control” would cost more over a 20-year period in human and financial terms than achieving eradication.”	[177]	
November-2007	“New studies in India and Nigeria showed that monovalent OPVs afford a 3-fold to 4-fold higher effectiveness per dose than trivalent OPV.”	[184,185]	
November-2008	“SAGE recommends that the mathematical model(s) of post-eradication risks be evaluated by Quantitative Immunization and Vaccine Related Research Advisory Committee (QUIVER).”		
April-2009	“Areas for further study include … models for estimating the risk of outbreaks of vaccine-derived polio in the post-eradication era, …. SAGE underlined the need for close interaction with QUIVER, given the relevance of its work to policy-making. SAGE must be fully briefed on critical assumptions and assured of the adequacy of data and methods for models that are used to inform policy decisions.” “The IPV working group presented a framework for evaluating post-eradication options for vaccination policy. SAGE was impressed with the work, but urged the working group to pay particular attention to uncertainties in mathematical modelling on the risks of emergence of vaccine-derived poliovirus.”		[182]
April-2010	“SAGE noted that the positive current epidemiological situation, the new strategic plan, and the sound economic argument for completing eradication together form a particularly appealing product for donors, warranting substantial further investment.”	[172]	
November-2010	Extended the term [for the SPWG] to “… allow the working group to benefit from considering … further mathematical modelling of post-eradication risks…” “SAGE expanded the working group’s remit by requesting it to assess whether, in view of the apparent eradication of type-2 wild poliovirus and the preponderance of circulating type-2 vaccine-derived polioviruses in recent years, trivalent OPV should be replaced with bivalent OPV for routine vaccination.”		
April-2012	“SAGE also received a report from the SAGE polio working group regarding a switch from trivalent oral poliovirus vaccine (tOPV) to bivalent OPV (bOPV types 1 and 3) and related policy and technical issues, and proposed recommendations for consideration by SAGE.”		[194]
April-2014	“Upon reviewing the relevant scientific evidence, SAGE endorsed updates to the existing WHO recommendations for travellers from polio-infected countries”		[195]
October-2014	“Lastly, SAGE endorsed the proposed risk-based approach for boosting immunity to type 2 poliovirus prior to OPV2 withdrawal, by ensuring that sufficient tOPV campaigns are planned and conducted to raise population immunity above the estimated threshold for transmission in areas at highest risk of cVDPV2 emergence. SAGE emphasized that planning for this risk-based approach should be done on a subnational basis.”		[191,196]
April-2015	“SAGE noted the increased scope of planned tOPV SIAs that will be implemented to reduce the risk of emergence of new cVDPV2, building on the risk-based approach endorsed by SAGE in October 2014. SAGE endorsed the proposed cVDPV2 elimination strategies in Nigeria and Pakistan and the programme’s risk-based approach to prevent and respond to new cVDPV2 emergence in any location.”		
October-2015	“The GPEI has optimized its strategy to prevent emergence of VDPV2 through an extensive set of tOPV campaigns, more sensitive definitions of cVDPV2, immediate response to any VDPV2 detection and updated its guidelines for responding to any cVDPV outbreak.”		[197] ^a^
April-2016	“SAGE reviewed the Polio Working Group discussion on future polio immunization policy. The Working Group proposed to work on the following recommendations: (i) an explicit decision on whether polio vaccination should be continued after global certification of eradication; (ii) the recommended IPV schedule (number of doses, timing, formulation) after OPV withdrawal; and (iii) the criteria for when countries could stop polio vaccination (e.g. surveillance capacity, absence of immunodeficiency-related vaccine-derived poliovirus), based on vaccine and funding availability and expected vaccine price”		
April-2017	“SAGE also reviewed the risk of reintroduction of polioviruses after global OPV cessation. The modelling and epidemiology suggest that VDPV may emerge 0–4 years after the global cessation of OPV use.”		^a,b^ [198] ^c^
October-2017	“SAGE acknowledged WHO’s work with Imperial College, London, to grade risks in Tier 3 and 4 countries based on susceptibility, transmission, exposure, and primary immunodeficiency-associated vaccine-derived poliovirus (iVDPV) prevalence.”	IC ^d^	
April-2019	“SAGE proposed that the GPEI determine the criteria for requesting that OPV2- containing vaccine production be resumed. SAGE agreed that discussions on the criteria are important and should be further explored during future working group meetings”		[199] ^e^
March-2020	“SAGE reviewed and agreed with the new GPEI strategy for responding to cVDPV2 outbreaks” (i.e., [160])… “SAGE recommended the strategy be more cautious about setting timelines for the introduction of nOPV2… [and] that tOPV be made available to countries for cVDPV2 outbreak response in subnational areas where there is co-circulation…. SAGE requested that GPEI further elaborate scenarios for using IPV in outbreak responses”		[68,200] ^a,b^
October-2020	“SAGE recommended that IPV should not be used for outbreak response because evidence demonstrates that IPV campaigns are unlikely to reach children not reached with OPV campaigns, have limited impact on stopping transmission and have a high programmatic cost. The priority of outbreak response is to stop transmission; therefore, activities should focus on rapidly achieving high coverage with OPV.”		[47]
March-2021	“SAGE acknowledged that countries are faced with complex decisions with regards to options for cVDPV2 outbreak response: should they use Sabin-based monovalent OPV type 2 (mOPV2) immediately and risk seeding new VDPV2s, or should they delay outbreak response until the country is programmatically prepared to use nOPV2? SAGE was presented with a modelling analysis of these options and agreed with the conclusion that countries facing cVDPV2 outbreaks should avoid delay and prioritize rapid, high-quality cVDPV2 outbreak response with whichever oral polio vaccine is available to them.”		[25]
October-2022	“SAGE was presented with a literature review and programme experience of using IPV for poliovirus outbreak control, and the role of IPV in preventing faecal-oral and oral-oral poliovirus transmission, as requested at the April 2022 SAGE meeting.”	[66]	[201,202] ^f^
March-2023	“SAGE reiterated its recommendation that outbreak responses be conducted without delay. For response using oral vaccines, nOPV2 should be preferred. However, mOPV2 could be used under exceptional conditions, e.g., if supplies of nOPV2 are inadequate, if emergency use listing (EUL) readiness cannot be achieved, and tOPV in the event of co-circulation of other poliovirus serotypes” “SAGE was presented with evidence of the role of IPV in areas of persistent poliovirus transmission”		[201,203,204] ^g^
September-2023	“Modelling analysis suggests that, if mOPV2 had been used instead of nOPV2 since March 2021, an estimated 43 new cVDPV2 emergences would have been detected by August 2023 compared with the 7 observed with nOPV2.”		^h,i,j^ [33] ^k^
February-2024	NA		[52,68] ^a^

Abbreviations: ACPE, Advisory Committee on Polio Eradication; BMGF, Bill and Melinda Gates Foundation; bOPV, bivalent OPV; cVDPV, circulating VDPV; EUL, emergency use listing; GPEI, Global Polio Eradication Initiative; IC, Imperial College; IDM, Institute for Disease Modeling; IPV, inactivated poliovirus vaccine; iVDPV, immunodeficiency-associated VDPV; KRI, Kid Risk, Inc.; LSHTM, London School of Hygiene and Tropical Medicine; mOPV, monovalent OPV; NA, not available; nOPV, novel OPV; OPV, oral poliovirus vaccine; OPV#, type # OPV (# = 1, 2, or 3); QUIVER, Quantitative Immunization and Vaccine Related Research Advisory Committee; SAGE, Strategic Advisory Group of Experts on immunization for the World Health Organization; SIA, supplemental immunization activity; SPWG, SAGE polio working group; tOPV, trivalent OPV; VAPP, vaccine-associated paralytic polio; VDPV, vaccine-derived poliovirus; VDPV#, type # VDPV (# = 1, 2, or 3); WHO, World Health Organization.

^a^ Presentation included unpublished work from IDM/BMGF (cited in some instances as Global Good, Intellectual Ventures, Institute of Disease Modeling, or IDM).

^b^ Unpublished work from IDM shows expected VDPV emergences each year for the first four years after global OPV cessation. Later reference in March 2020 refers to lower unpublished expected numbers than provided by IDM in 2017, with attribution of the lower numbers to the GPEI Eradication and Outbreak Management Group in a table that shows comparisons between pre-OPV2 cessation predictions and post-OPV2 cessation epidemiological experience.

^c^ One presentation discussed the future immunization policy for IPV.

^d^ Unpublished work by IC.

^e^ One presentation discussed new guidelines for surveillance of patients with primary immunodeficiency disorders [189], although this topic did not appear in the conclusions and recommendations from the meeting.

^f^ One presentation on the role of older age groups in transmission did not mention previously-published relevant modeling studies [1] or a study showing the need to target birth cohorts born since 2016 now above age 5 years of age for type 2 oSIAs [34].

^g^ The evidence of the role of IPV did not include relevant studies published by KRI.

^h^ The modeling mentioned in the conclusions and recommendations provided a reference to a conference abstract for modeling performed by IDM.

^i^ Publications relevant to the failure of OPV2 cessation by KRI not discussed include [7,8,12,15,36].

^j^ One presentation discussed outbreak responses to cocirculation that did not include relevant published modeling that did not support the recommendations made by SAGE at the meeting [35].

^k^ One presentation included modeling related to the certification of WPV1 [33], although this topic did not appear in the conclusions and recommendations from the meeting.

**Table 5:** Representation of modeling at face-to-face SAGE polio workgroup meetings.

Meeting		Roles ^a^				Information Extracted from the Note for the Record	
N	Date	KRI	IC	LSHTM	IDM/BMGF	Presentation Topic (Presenting Modeling Group)	Group [Source(s)]
1	October-08	M *	M *			Modeling the risks: past and future (KRI) Modeling: The next frontiers (IC)	KRI [183]
2	June-09	M	M	P *		Cost-effectiveness of routine polio vaccination (LSHTM) Poliovirus transmission potential (LSHTM)	KRI [177,181,182,183,194,205,206,207,208], LSHTM [107], IC [185,186]
3	March-11	M *	M		P *	Modeling cVDPV risks/post-eradication policies (KRI) Options, risks, and prerequisites for OPV2 cessation (KRI) Modeling tools for cVDPV emergence (IDM)	
4	February-12	M *	M *		P *	Wide-spread transmission of type 2 cVDPV in Nigeria (IC) cVDPV emergence risks pre- and post-eradication (IDM) Modeling and managing VDPV risks: known and not (KRI)	NA
5	November-12	M	M				
6	June-13	M	M		P *	VDPV emergence risk for mOPV2 post-OPV2 cessation (IDM)	NA
7	October-13	M	M *		P *	Post-cessation outbreak response, OPV use, cVDPV risk (IDM)	
8	February-14	M	M *			Review of the duration of mucosal immunity to poliovirus (IC)	
9	July-14	M *	M *		P *	Modeling the risk of cVDPV emergence (KRI) Risk of VDPV emergence and spread (IC) Non-polio-AFP population immunity projections (IDM) tOPV campaigns pre-OPV2 cessation (IDM)	KRI [196,209,210] IC ^b^
10	September-15	M	M *		P	Serotype 2 vaccine-derived poliovirus risk assessment (IC)	
11	January-16	M ^	M ^		P	Type 2 outbreak protocol ^c^ Risks of cVDPV emergence, based on modeling (IDM)	
12	August-16	M *	M *		P *	Detection of type 2 Sabin virus after the switch (IC) Needs for bOPV campaigns prior to OPV13 withdrawal (KRI) Assessment of risks and implications of bOPV use (IDM)	
13	February-17	M *	M *		P *	Roles of different vaccines in outbreaks and the endgame (KRI) Polio endgame modeling, IPV in SIAs (IDM) Impact of IPV in RI and SIA following OPV2 withdrawal (IC)	
14	September-17	M	M		P *	OPV13 cessation and SIA planning (IDM)	
15	February-18		M		M *	Risk assessment for bOPV cessation (IDM), Role of bOPV preventive SIAs pre-bOPV cessation ^d^	
16	September-18		M *	R	M	Country prioritization of IPV catchup immunization (IC)	
17	February-19		M	R	M	Risks and the role of bOPV preventive SIAs pre-cessation ^d^	
18	September-19		M	P *	M	VAPP analysis (LSHTM)	
19	February-20		M *	P *	M	Role of IPV for outbreak response (IC), Scenarios for initial nOPV2 use under EUL (LSHTM)	
20	September-20		P *	M *	M *	Impacts of polio eradication activity disruption (LSHTM) ^e^ IPV use for cVDPV and WPV outbreak response (IC) Tracing infections and micro vaccination campaigns (IDM)	
21	February-21		P *	M *	M	nOPV2 versus mOPV2 for outbreak response (IC) Transition from initial to wider nOPV2 use criteria (LSHTM)	
22	August-21			M *	M	Update on nOPV2 policy (LSHTM)	
23	March-22		P *	M *	M	Field seroprevalence and immunogenicity studies (LSHTM) nOPV2 effectiveness study (IC)	
24	August-22			M *	M	Review of data on use of IPV for outbreak response (LSHTM)	
25	March-23			M *	M	Update on iVDPV epidemiology (LSHTM)	
26	August-23			M *	M	Options for type 1 and 2 co-circulation outbreaks (LSHTM)	
27	February-24			M	M	bOPV cessation planning summary of modeling ^f^	

Abbreviations: AFP, acute flaccid paralysis; BMGF, Bill and Melinda Gates Foundation; bOPV, bivalent OPV; cVDPV, circulating VDPV; EUL, emergency use listing; GPEI, Global Polio Eradication Initiative; IC, Imperial College; IDM, Institute for Disease Modeling; IPV, inactivated poliovirus vaccine; iVDPV, immunodeficiency-associated VDPV; KRI, Kid Risk, Inc.; LSHTM, London School of Hygiene and Tropical Medicine; mOPV, monovalent OPV; NA, not available; nOPV, novel OPV; OPV, oral poliovirus vaccine; OPV#, type # OPV (# = 1, 2, or 3); RI, routine immunization; SAGE, Strategic Advisory Group of Experts on immunization for the World Health Organization; SC, stochastic compartmental; SIA, supplemental immunization activity; SPWG, SAGE polio working group; tOPV, trivalent OPV; VAPP, vaccine-associated paralytic polio; VDPV, vaccine-derived poliovirus.

^a^ M indicates attended as a SPWG member, P indicates invited external participants, R indicates rapporteur, and * indicates presented at the meeting.

^b^ Also referred to an unpublished IDM analysis and an IC study ultimately published in 2016 [211].

^c^ Presentation included modeling from KRI, IC, and IDM developed following a pre-meeting in-depth modeling discussion.

^d^ Presentation that referred to KRI work.

^e^ Presentation that referred to KRI, IC, IDM, and LSHTM work.

^f^ Presentation that referred to KRI, IC, IDM/BMGF, and LSHTM work.

## Abbreviations

ACPE, Advisory Committee on Polio Eradication; AFP, acute flaccid paralysis; BMGF, Bill and Melinda Gates Foundation; bOPV, bivalent OPV; cVDPV, circulating VDPV; DEB, differential equation-based; DES, discrete event simulation; EUL, emergency use listing; GIT, Georgia Institute of Technology; GPEI, Global Polio Eradication Initiative; IB, individual-based; IC, Imperial College; IDM, Institute for Disease Modeling; IPV, inactivated poliovirus vaccine; iVDPV, immunodeficiency-associated VDPV; KRI, Kid Risk, Inc.; LPV, live poliovirus; LSHTM, London School of Hygiene and Tropical Medicine; mOPV, monovalent OPV; NA, not available; nOPV, novel OPV; OPV, oral poliovirus vaccine; OPV#, type # OPV (# = 1, 2, or 3); oSIA, outbreak response SIA; PRC, GPEI polio research committee; pSIA, preventive SIA; RI, routine immunization; QUIVER, Quantitative Immunization and Vaccine Related Research Advisory Committee; SACEMA, the South African Centre for Epidemiological Modelling and Analysis; SAGE, Strategic Advisory Group of Experts on immunization for the World Health Organization; SC, stochastic compartmental; SIA, supplemental immunization activity; SPWG, SAGE polio working group; tOPV, trivalent OPV; VAPP, vaccine-associated paralytic polio; VDPV, vaccine-derived poliovirus; VICP, Vaccine Injury Compensation Program (United States); WHA, World Health Assembly; WHO, World Health Organization; WPV, wild poliovirus; WPV#, type # WPV (# = 1, 2, or 3).
